# Popular media records reveal multi-decadal trends in recreational fishing catch rates

**DOI:** 10.1371/journal.pone.0182345

**Published:** 2017-08-04

**Authors:** Ruth H. Thurstan, Edward Game, John M. Pandolfi

**Affiliations:** 1 School of Life and Environmental Sciences, Centre for Integrative Ecology, Deakin University, Warrnambool, Victoria, Australia; 2 Australian Research Council Centre of Excellence for Coral Reef Studies and School of Biological Sciences, The University of Queensland, St Lucia, Queensland, Australia; 3 The Nature Conservancy, Conservation Science, South Brisbane, Queensland, Australia; 4 Biodiversity and Conservation Science Centre, The University of Queensland, St Lucia, Queensland, Australia; Leibniz Centre for Tropical Marine Research, GERMANY

## Abstract

Despite threats to human wellbeing from ecological degradation, public engagement with this issue remains at low levels. However, studies have shown that crafting messages to resonate with people’s personal experiences can enhance engagement. Recreational fishing is one of the principal ways in which people interact with aquatic environments, but long-term data from this perspective are considered rare. We uncovered 852 popular media records of recreational fishing for an Australian estuary across a 140-year period. Using information contained in these articles we analysed the species composition of recreational catches over time and constructed two distinct time series of catch and effort (n fish fisher^-1^ trip^-1^; kg fish fisher^-1^ trip^-1^) for recreational fishing trips and fishing club competitions (mean n and kg fish caught across all competitors, and n and kg fish caught by the competition winner). Reported species composition remained similar over time. Catch rates reported from recreational fishing trips (1900–1998) displayed a significant decline, averaging 32.5 fish fisher^-1^ trip^-1^ prior to 1960, and 18.8 fish fisher^-1^ trip^-1^ post-1960. Mean n fish fisher^-1^ competition^-1^ (1913–1983) also significantly declined, but best n fish fisher^-1^ competition^-1^ (1925–1980) displayed no significant change, averaging 31.2 fish fisher^-1^ competition^-1^ over the time series. Mean and best kg fish fisher^-1^ competition^-1^ trends also displayed no significant change, averaging 4.2 and 9.9 kg fisher^-1^ competition^-1^, respectively. These variable trends suggest that while some fishers experienced diminishing returns in this region over the last few decades, the most skilled inshore fishers were able to maintain their catch rates, highlighting the difficulties inherent in crafting conservation messages that will resonate with all sections of a community. Despite these challenges, this research demonstrates that popular media sources can provide multiple long-term trends at spatial scales, in units and via a recreational experience that many people can relate to.

## Introduction

Scientists have identified ecosystem degradation as an urgent and global threat, yet public engagement with this issue remains at low levels [1]. A growing body of literature explores the social and cultural reasons why scientific warnings about impending environmental crises commonly fail to engage the public, and how this situation can be reversed [1,2]. Studies have shown that factors such as inspiring a connection to nature and crafting messages that resonate with peoples’ personal experience or sense of place, among others, are required to cultivate environmental stewardship [1,3]. Therefore, studies that frame ecological change at the local scale, as a tangible problem, and as personally relevant may ultimately be more successful at stimulating engagement than examples framed at a national or global scale [3].

Compounding the problem of public engagement is that, in many locations, ecological degradation commenced decades or even centuries prior to scientific monitoring [4,5]. Long-term degradation, coupled with a lack of data on past ecological states results in intergenerational shifts in our expectation of what is a ‘natural’ or ‘healthy’ ecosystem, known as the shifting baseline syndrome [6]. Consequently, there is a need to identify data sources that improve our understanding of the magnitude of change in the years prior to formal data collection.

Worldwide, millions of people take part in recreational fishing activities every year, and in some inshore regions recreational fishers harvest greater quantities of fish than the commercial sector [7–9]. Recreational fishers have been implicated in ecological degradation [10,11], but have also been enthusiastic supporters of conservation and research agendas that otherwise would have experienced little public engagement [12]. The long history of recreational fishing and high levels of participation in many regions provide an opportunity not only to fill gaps in our knowledge of coastal marine ecosystem trends, but to explore changes anchored at a local scale, using metrics and a cultural experience that large numbers of the public can relate to. However, systematic data collection is usually lacking for recreational fisheries [10], and researchers have thus turned to alternative sources of data to evaluate catch and size trends. These include fishing club data [13,14], personal diaries [15], logbook records [16], magazines [17] and photographs [18]. However, many of these sources are not commonly available and access may be restricted (e.g., fishing club records, logbooks), or comparable data through time are rare (e.g., photographs).

We examined an open-access and widely available historical data source, newspapers, from the 19^th^ century onwards, to evaluate what information existed on recreational resource use and ecosystem change over time. We focused our search at an estuary scale, the Noosa Estuary in Queensland, Australia. Quantitative data contained in these media articles enabled us to construct several distinct recreational catch rate trends up to 98-years in length, representing some of the longest recreational time series uncovered to date. We demonstrate that information on coastal resource use exist in abundance within popular media archives, and discuss how these data can contribute to scientific and public understanding of ecosystem change.

## Methods and materials

### Study site

**Fig 1:**
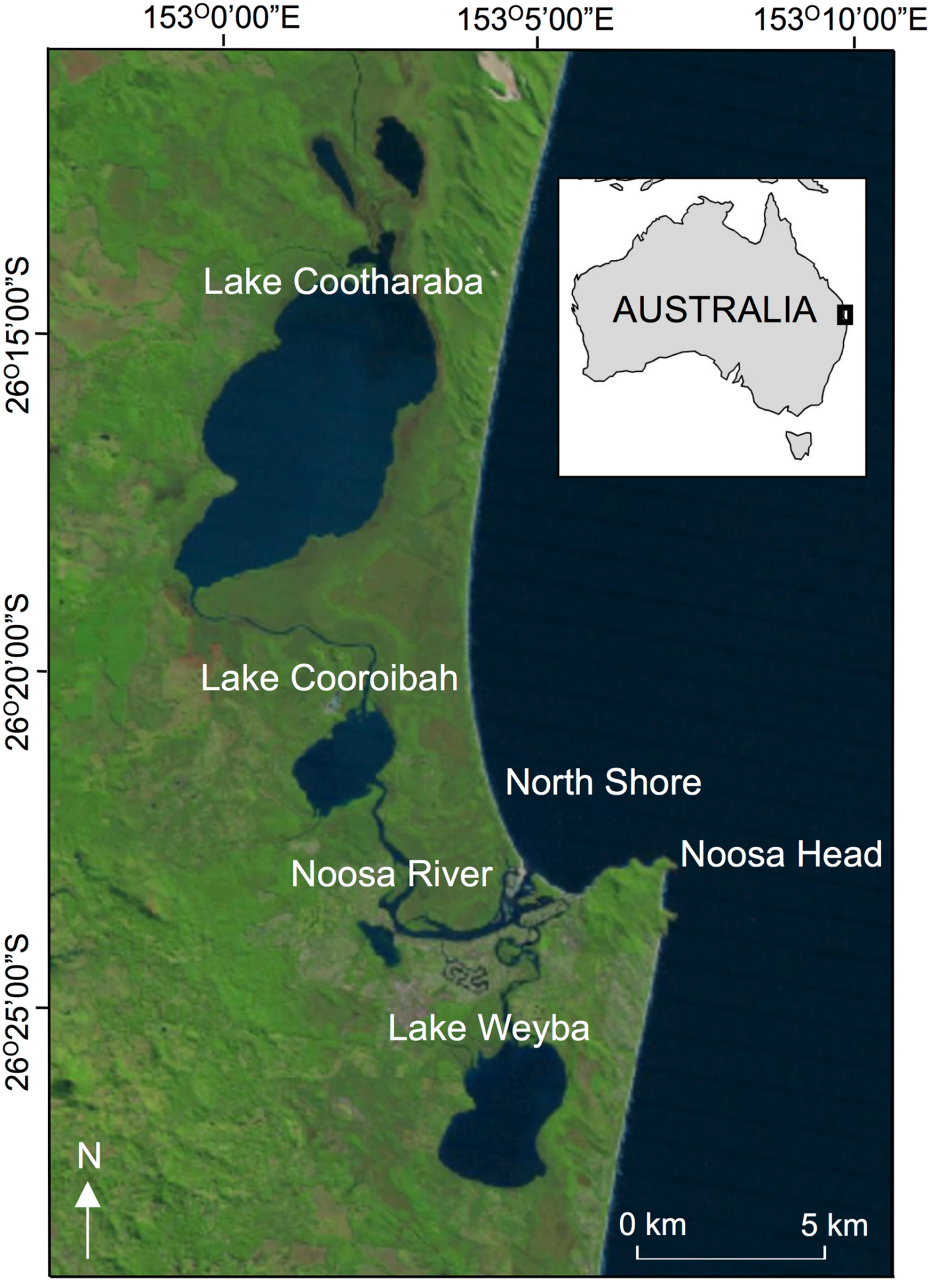
Map showing the Noosa Estuary and locations mentioned in the text. Basemap sourced from the Department of the Interior/U.S. Geological Survey.

The Noosa Estuary is situated in southeast Queensland, Australia (Fig 1). The estuary comprises a tidal head <30km in length [19]. It is a shallow (generally <3m) subtropical estuarine system mixed primarily by tidal currents, although wind also drives the mixing of a series of wide, shallow embayments, the ‘Noosa Lakes’. Oceanic exchange is restricted due to the presence of a shallow sand bar (<2m) at the mouth of the river [20]. The upper catchment of the river is largely composed of natural forest, while the lower system has been heavily modified throughout the late 20^th^ century as a result of residential canal development and shoreline protection [19]. For this study, we defined the geographical limits of the estuary as the saltwater river system and included the saltwater lakes (Weyba, Cootharaba and Cooroibah Lakes), Noosa Head and the North Shore (ocean) beach if it was stated that fish were caught near the mouth of the estuary (Fig 1).

### Data sources

We sourced materials from local and state libraries, in addition to the National Library of Australia’s digitised collections [21] and government archives (S1 Table). Digitised material was searched using a variety of keywords to try to ensure that the maximum number of records were made available. These included key locations, e.g., “Noosa” AND “fish*”, “Tewantin” AND “fish*”, “Weyba” AND “fish*”, “Cootharaba” AND “fish*”, “Cooroibah” AND “fish*”, and older spellings e.g., “Newsa” AND “fish*”. Additional search terms were also conducted with the aim to identify any wider environmental or social changes that might aid data interpretation e.g., “Tewantin” AND “oyster”, “fishing” AND “competition” AND “rules”. Archival material that was not keyword searchable was examined using either microfiche or hard copies of publications. We collated information on locations fished, species caught and narratives or correspondence related to fishing in the Noosa Estuary, distinguishing between recreational fishing trips and club-affiliated fishing competitions, and excluding fishing activities that took place in the open ocean away from the estuary and adjacent beaches, or which used fishing nets rather than lines. Line and net fishing activities were usually clearly distinguished in the popular media, but to ensure that different fishing methods were not erroneously compared, species deemed unlikely to take a hook, or activities that were not strictly line fishing (e.g., the catching of fish by ‘jagging’, whereby sharpened hooks are thrown at the school of fish on the surface and fish caught by being hooked in the body), were discarded from analysis. We extracted quantitative data, for example, number of fish caught, number of people fishing, number of hours fished, where available.

### Data analysis—Species composition

To analyse changes in catch composition over time we conducted a one-way Analysis of Similarities (ANOSIM) test. ANOSIM compares the degree of dissimilarity within and between groups; in this case, we used decade as our sampling unit. As species were most commonly reported as present, rather than by quantity, similarities were measured using the Jaccard Index of Association, which is commonly applied to binary data [22]. We made the assumption that if a species was not mentioned in the catch record, it had not been caught. Decades with <10 data points were not included in the analysis. Data were analysed using the Vegan package in R [23,24].

### Data analysis—Catch rates

We calculated catch rates using three metrics, based on data availability: number of fish caught fisher^-1^ trip^-1^, kg of fish caught fisher^-1^ trip^-1^, and number of fish caught fisher^-1^ hour^-1^. Three separate time series were calculated: recreational catch rates (trips not affiliated with fishing club activity), best competitive catch rate (the greatest number or weight of fish caught by an individual club member during a fishing competition), and mean competitive catch rate (the number or weight of fish weighed in after a club competition, divided by the number of people reported fishing during the competition).

The majority of recreational data points fell into two distinct periods, 1920–1945 and 1970–1985, hence we split this dataset into two groups prior to analysis: catch rates recorded pre-1960 and catch rates recorded post-1960. Club records were more evenly spread throughout the time series, hence we conducted a linear mixed effect analysis on the four fishing club time series using the lmer function in the lme4 package in R [25]. To calculate the relationship between catch rate (n or kg) and year, we entered catch rate into the model as a response term, year as a fixed effect, and fishing club as a random effect. Catch rates were transformed prior to analysis using the Box-Cox transformation. We undertook likelihood ratio tests of the full model against a null model that excluded the effect of year to calculate p-values [26].

We also sourced catch rates from a government survey. These could not be compared to newspaper records of numbers or weight of fish per trip as government data were usually obtained by inspection of recreational fishers’ catches in the midst of their fishing activities. However, numbers of fish, people actively fishing and hours fished were recorded by the inspectors, enabling comparisons between recreational catch fisher^-1^ hour^-1^ and government data. Inspectors also noted down the number of fish that were landed but below minimum size regulations.

We used the Kruskal-Wallis one-way analysis of variance to determine whether statistically significant differences existed among multiple datasets, and conducted post-hoc tests using Dunn’s multiple comparisons test. We used the Mann-Whitney test for comparisons between two groups.

## Results

We searched approximately 10,000 records from government reports and popular media for records of fishing activity in the Noosa Estuary. From these sources we extracted 852 records (qualitative and quantitative) on recreational fishing, spanning the years 1873–2014. A further 98 records of recreational fish catches were sourced from government inspections occurring in 1962 and 1963. No government inspection data were found for subsequent years.

### Species composition

291 records provided information on species included in the catch. All records were extracted from the recreational time series, as competition records rarely reported the species of fish caught. Fishers reported catching a variety of species; of which the most commonly mentioned were bream (*Acanthopagrus australis* and *Rhabdosargus sarba*; mentioned as present in 50% of species-specific records), flathead (*Platycephalus* spp; 45% of records), whiting (*Sillago* spp; 40% of records), tailor (*Pomatomus saltatrix*; 32% of records), and mulloway (*Argyrosomus japonicas*; 16% of records). These species remained the most frequently reported over time, although their percentage occurrence varied by decade (S1 Fig). ANOSIM test results provided an overall R statistic of 0.135 (p = 0.001). The closeness of the R statistic to zero indicates a lack of separation among decades (i.e., species composition within and between decades is similar) and thus signifies no major shift in species reported as present in the catch over time [27].

### Recreational trips

**Fig 2:**
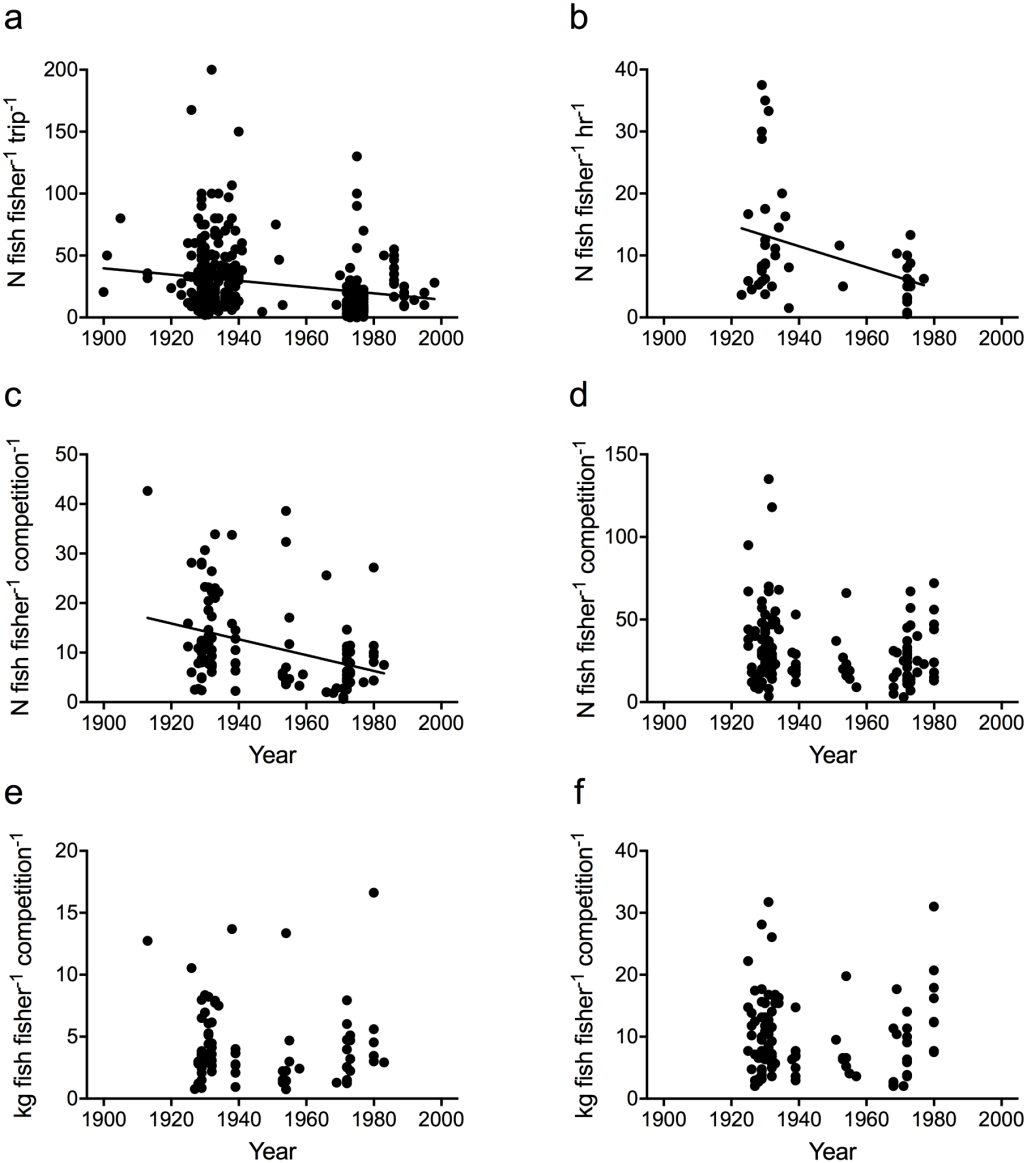
Catch rates sourced from popular media. Catch rates recorded from recreational fishing trips (A and B) and fishing club competitions (C to F). A) Number of fish caught fisher^-1^ trip^-1^ (n = 340; linear regression, y = −0.2524*x + 519.2; p = 0.0001); B) Number of fish caught fisher^-1^ hour^-1^ (n = 48; linear regression, y = −0.1702*x + 341.7; p = 0.0081). C) Mean number of fish caught fisher^-1^ competition^-1^ (n = 112; mixed model estimate, -0.003; p = 0.001); D) Number of fish caught by the winning fisher (n = 134; mixed model estimate, -0.001; p = 0.192). E) Mean weight of fish caught fisher^-1^ competition^-1^ (n = 82; mixed model estimate, -0.0001; p = 0.226); F) Weight of fish caught by the winning fisher (n = 105; mixed model estimate, -0.0002; p = 0.724).

Results of recreational (non-club) fishing trips were quantitatively described in local and regional newspapers between 1900 and 1998 (Table 1), enabling us to calculate catch rates (n fish fisher^-1^ trip^-1^; n = 340) over this period. Trends in weight of fish caught could not be calculated for recreational trips as weights were recorded infrequently and using inconsistent metrics (e.g., average weight, heaviest fish or total weight of fish in the catch). Early n fish fisher^-1^ trip^-1^ were significantly higher than catch rates recorded in the later period (Mann-Whitney U = 7630.0; p = <0.0001; Fig 2A), with a mean value of 32.5 fish fisher^-1^ trip^-1^ (SD = 1.8) pre-1960, and 18.8 fish fisher^-1^ trip^-1^ (SD = 1.9) post-1960 (42% decline; Fig 2A). Catch rates recorded as n fish fisher^-1^ hour^-1^ (n = 48) also displayed a significant decline with time, with a mean catch rate of 13.0 fish fisher^-1^ hour^-1^ (SD = 1.7) pre-1960, and 5.9 fish fisher^-1^ hour^-1^ (SD = 0.9) post-1960 (Mann-Whitney U = 125.0; p = <0.0056; Fig 2B). Of these 340 recreational fishing trips, 60 (18%) explicitly stated that the fishers were visitors to the Noosa region.

**Table 1:** Examples of recreational fishing results recorded in newspapers, with quantitative data used to evaluate catch rates highlighted in bold.

Quote	Source
“**Wednesday was spent on the river under the experienced guidance of one of our oldest fishermen, and his Excellency and Captain Pennefather showed their proficiency in this branch of sport by bringing home the good basket of 28 taylor, 12 bream, and one trevally**.”	Gympie Times, 21 Jul 1900
“**A party [6 names] journeyed by the motor launch Sunrise from Cootharaba Lakes to Noosa Heads on Oct 31. The total catch numbered 142 fish. The fish were secured from the beach in the channel at the mouth of Noosa River**.”	Daily Standard, 12 Nov 1920
“**E. A. Osborne and H. W. Page, of Pomona, spent a few hours' fishing, catching 71 fish, of which 42 were nice sized flathead**.”	Sunday Mail, 15 Dec 1929
“**A competition was also held on the night of the 16th inst., […] winner 34 fish, total number of fish caught for three hours fishing was 146, with 13 members competing**.”	Maryborough Chronicle, 26 Oct 1925
“**The Cooroy Fishing club conducted a competition. 17 competitors weighing in 316 fish, weighing 227.5 lb. A. Gibson won with a catch of 70 fish**.”	The Brisbane Courier, 4 Aug 1931

The number of hours reported fishing during recreational trips showed no significant change between the two periods (n = 48, Mann-Whitney U = 215.5; p = 0.4617) and were highly variable, averaging 3.6 hours per trip (SD = 4.1).

### Fishing club competitions

Catch rates from club competitions were reported in newspapers from 1913 to 1983 (mean, 1913–1983; best, 1925–1980). Significant declines were observed in mean n fish fisher^-1^ competition^-1^ over time (mixed model estimate = −0.003, p = 0.001, Fig 2C, S2 Table). No significant change was observed in best n fish fisher^-1^ competition^-1^ (mixed model estimate = −0.001, p = 0.192, Fig 2D, S2 Table), mean kg fish fisher^-1^ competition^-1^ (mixed model estimate = −0.0001, p = 0.226, Fig 2E, S2 Table), or best kg fish fisher^-1^ competition^-1^ (mixed model estimate = −0.0002, p = 0.724, Fig 2F, S2 Table). Mean catch rates averaged 11.2 fish fisher^-1^ competition^-1^ (SD = 7.9) during the 1920s, declining to 7.2 fish fisher^-1^ competition^-1^ (SD = 4.9) in the 1970s and 80s (36% decline). Best catch rates averaged 31.2 fish fisher^-1^ competition^-1^ (SD = 21.1) throughout the time series. Mean weights averaged 4.2 kg fisher^-1^ competition^-1^ (SD = 3.1), while best weights averaged 9.9 kg fisher^-1^ competition^-1^ (SD = 6.2) throughout the time series.

Although data were limited (n = 32) a significant increase in hours fished with time was observed for fishing competitions (linear regression, y = 0.1796*x - 342.4, p = 0.0001). Hours fished averaged 3.8 hours during the 1920s (SD = 2.3, n = 5), and 11.7 hours (SD = 4.4, n = 21) by the 1970s.

### Government records

Catch rates recorded by government officials averaged 2.3 (SD = 3.1) fish fisher^-1^ hour^-1^ throughout the 1962–63 period. Of the fish inspected, 25% were found to be below the minimum size limit.

### Time series comparisons

Statistically significant differences existed among the recreational, mean and best competition time series (Kruskal-Wallis statistic = 99.77, p = <0.0001). Post hoc tests revealed that mean competition catch rates (n fish fisher^-1^) were significantly lower than both recreational (mean rank difference = 153.2; adjusted p = <0.0001) and best competition catch rates (mean rank difference = 207.8; adjusted p = <0.0001). Best competition catch rates were also significantly higher than recreational catch rates, although these two time series demonstrated the least difference (mean rank difference = 54.61; adjusted p = 0.0046).

Comparisons of recreational n fisher^-1^ hour^-1^ with comparable government data showed that recreational catch rates were significantly higher than catch rates as recorded by government officials (Mann-Whitney U = 480.5; p = <0.0001).

## Discussion

In this study we demonstrated that information on recreational fishing trips and competitions, including nearly a century of catch and effort data, exist within newspaper archives, contributing to our understanding of ecological change, historical resource use and recreational activities in coastal seas. Moreover, this widely available data source provides information at a local scale, using an experience and units that are readily identifiable to members of the public [3].

### Interpretation of catch rate trends

Despite the Noosa Estuary not being picked for its data availability, to our knowledge the time series we constructed are some of the longest recreational fishing time-series data collated at an estuary scale. These time series reveal differing trends among fishing groups and metrics (n or kg) analysed. N fish fisher^-1^ declined significantly in recreational and mean fishing club competition records over time, while n fish fisher^-1^ caught by competition winners displayed no significant trend. The weight of fish caught during competitions, both mean and winning catch, showed no significant change over time.

Shifts in targeting behaviour, either through changing angler preference for particular species, regulatory changes or attitudinal shifts, may confound interpretations of catch rate trends over time. While there appears to be discrepancies among the trends observed in mean competition n fish fisher^-1^ and kg fish fisher^-1^, these differences may be due to competition anglers purposefully targeting larger fish over time. This cannot be directly tested due to a lack of individual size data. However, minimum landing sizes for popular recreational species groups (e.g., bream, whiting, flathead) were gradually increased during the early 20^th^ century (e.g., The Fish and Oyster Act of 1914; Amendment 1926), and historical records demonstrate that minimum landing sizes were policed during fishing competitions (The Telegraph, 18 May 1936), while clubs sometimes imposed minimum size limits that were more restrictive than government regulations (Daily Standard, 26 Jul 1918). Moreover, the majority of competitions in our sample scored fishers by points, which were awarded based on a combination of the number and weight of fish caught (The Telegraph, 26 Nov 1920; 15 Jun 1936). Hence, there were incentives during competitions to catch both as many and as heavy a fish as possible.

The observed declines in n fish fisher^-1^ trip^-1^ observed in the recreational time series is, however, unlikely to be due to shifts in targeting, as the most commonly reported species remained the same over time. The changing frequency of occurrence of particular species observed in our dataset could be related to species-specific targeting shifts (S1 Fig), but additional data would be required to test this hypothesis. Although minimum landing sizes increased incrementally over time for some recreationally targeted species, we suggest that these regulations had little impact upon non-competitive recreational fishers’ catches or catch rates. Available government data suggest a broad disregard or ignorance by recreational fishers of minimum landing sizes. Likewise, in-possession limits are likely to have had a limited impact on either recreational or competition catch rates, as these did not come into force until towards the end of the time series (around 1990) and were not imposed on all species.

Changing attitudes among recreational fishers and fishing column writers, and the potential impact of attitudinal shifts on the frequency of reporting of large catches over time, must also be considered. Towards the end of the time series fishing columnists increasingly rewarded the capture of large individual specimens rather than reporting numbers of fish caught (e.g., Noosa News 3 Jan 1986). Despite this shift, large catches of fish appeared to remain socially acceptable, and were reported upon, into the 1990s. It was only at the end of the time series that methods such as catch and release began to be increasingly highlighted [28], reflecting a shift away from the ‘take-all’ mentality that dominated throughout the 20^th^ century. Likewise, fishing clubs continued to reward the largest number of fish caught until the end of the time series (e.g., Noosa News, 25 Aug 1977), although sport fishing clubs, which rewarded the diversity of catch and weight of individual fish rather than quantity, became increasingly popular from the 1970s onwards (e.g., Noosa News 5 May 1972).

Catch rate trends may also have been influenced by increasing numbers of fishers. While we were able to generate catch rate trends for individual fishing trips from popular media reports, we found no information on the total quantity of fish caught by recreational fishers, or an indication of trends in the number of people recreationally fishing over the years. It is likely that numbers of recreational fishers have significantly increased, leaving open the possibility that declining catch rates are a reflection of the same total catch being shared among more fishers. While increasing fisher numbers could potentially explain the declining trends, we cannot test this using the available data. Moreover, individual fishing effort over this period increased, which would conversely work to mask catch rate declines. For example, over the time series, fishing competitions increased the time available to competitors to fish, while improvements in fishing techniques and an increase in available fishing ground is also likely to have occurred, as clubs increasingly arranged trips to correspond with favourable tides, standardised and improved their fishing gear, and upgraded rowing boats to motor boats [13].

Finally, wider ecosystem change in the Noosa Estuary may also have impacted catch rates by reducing the system’s ability to support the same quantity or diversity of fish. For example, the lower reaches of the Noosa Estuary have been subject to heavy development pressure since the mid to late 20^th^ century [19]. Coastal development has resulted in substantial narrowing of the river mouth, and the loss of previously productive nursery and fishing areas [29,30]. Archival records also reveal a once highly productive oyster fishery within the Noosa Estuary, which had ceased to exist by the mid 20^th^ century [31].

The data from popular media and government archives are limited in that they do not allow us to test the effects of all the potential social and ecological changes that may have occurred. However, the available evidence, that club members who won competitions often succeeded by a large margin and had significantly higher catch rates compared to other club and recreational fishers, alongside the qualitative information that suggests a proportion of the recreational fishers reporting their catches were visitors to the area, suggests that recreational fishers with superior fishing skills and local knowledge were able to maintain catch rates while less skilled or less knowledgable recreational fishers’ catch rates declined.

### Comparability of popular media to other historical sources

The different record types also enables comparison of popular media with government records. Recreational catch per hour was significantly higher than government data, suggesting that newspaper reports were biased towards reporting the best recreational catches. This is likely to have little influence on how we interpret the trends observed unless we have reason to believe media reporting bias to significantly alter over time. However, it does indicate that these catch rates should not be directly compared to other forms of recreational fishing data (e.g., data from fishing surveys).

Data derived directly from fishing clubs are also useful in gauging the accuracy of popular media accounts. We were unable to source records directly from local fishing clubs for this study, but previous studies have evaluated catch rate trends in wider southeast Queensland from fishing club data. Pollock and Williams [13,32] assessed fishing club data over 35 years (1945–1980) for surf fisheries in Moreton Bay, approximately 80 km south of our study area. These data were of sufficient resolution to depict seasonal trends, as well as species-specific trends in catch rate. Although analyses indicated an increase in catch rates for particular species (whiting and sea bream during spawning season), which the authors assumed were due to improvements in fishing techniques, no significant changes in the catch rate of mixed fish were observed over time [13]. A study conducted 10 years later in the same region reported no evidence of overall change in the catch rate of whiting in the Moreton Bay region, although localised differences in trends occurred [33]. Conversely, a study of fishing club catch rates from inshore locations around Gladstone (situated to the north of our study area) showed significant declines in catch rates of total fish caught between 1982 and 2001 [34].

The differences observed between fishing club record trends and the trends highlighted in popular media may be due to the different geographical locations, or the differing temporal period over which data are available. In the newspaper records, some of the highest recorded catch rates occurred during the 1920s and 30s, a period not covered by the published fishing club records. While available fishing club records provide catch rates to a resolution (species-specific, seasonal) that popular media does not, such data are not always accessible: over time records may be lost or access limited due to club members’ concerns over the use of their data. Conversely, popular media records are open access and widely available, and not limited to a particular region or specific club’s activities.

### Implications of findings for conservation

Recreational fishers—particularly those from the same region—are often characterised as a single stakeholder group for conservation and management purposes. However, the different catch rate trends observed suggest that fishers of varying skill levels are likely to have distinctly different experiences of fishing, even when targeting the same fishing grounds. This in turn, is likely to result in varied perspectives on the magnitude of change in fish availability and abundance over time. Different experiences and perspectives not only have ramifications for effectively engaging stakeholders in conservation, but also how we interpret resource users’ (potentially highly variable) accounts of long-term ecological change, which is a focus of increasing research interest to natural scientists [35,36]. The upward bias observed in newspaper reports compared to government records also highlights the complexities of interpreting data sourced from archival records that were not recorded for the purposes of examining resource use or ecological patterns over time. This complexity is compounded in popular media sources, as the reasons for reporting particular fishing activities may not just be related to the availability of that information, but also a response to factors such as reporting preference and style, changing societal norms and/or change to regulations, among other factors [37].

Despite the complexity of interpreting trends from popular media, if interpreted with care they have the potential to fill major gaps in our knowledge of ecological change and historical resource use using scales, units and an activity that many people can relate to. In this case, these recreational fishing trends, alongside qualitative information on estuarine changes resulting from land-use practises, coastal development and removal of oyster habitat, were presented to members of the Noosa Council in 2016. These findings contributed to a decision by Council and partner organisations to fund a $1 million (AUD) trial to increase marine diversity and fish abundance in the Noosa Estuary [38].

Many coastal marine ecosystems have been heavily impacted by human activities over long periods, but our awareness of long-term change is masked by short-term environmental variability, the prevalence of the shifting baseline syndrome, and lack of data [4,6]. Together, these present significant barriers to effective public engagement. While our time series cannot directly interpret changes to coastal fish populations, they reveal a century of diminishing returns to all but the most skilled or dedicated of recreational fishers. Describing long-term ecosystem change from this perspective refocuses this issue from the global and abstract, to a local and tangible problem. Demonstrating this loss of experience to recreational users and the local community thus provides an opportunity to foster awareness of some of the more pervasive impacts of long-term ecological change. Furthermore, it sends the message that ecological degradation impacts people even when they don’t depend upon this ecosystem for their livelihoods. The growing availability of open source historical data means that this research is likely to be replicable across multiple ecosystems, and for any location where a cultural tradition of recreational resource use exists.

## Supporting information

S1 Fig Percentage occurrence of species as mentioned in catch records. Percentage of catch records reporting to species that mentioned the occurrence of either whiting, bream, flathead, mulloway, tailor or other species. Decades where <10 records mentioned specific species in their catch are not included.
(TIFF)

S1 Table Sources searched for archival material on the Noosa Estuary. (PDF)

S2 Table Results of linear mixed model analyses for temporal trends in fishing club competitions within the Noosa Estuary. (PDF)
