# Supplementary figures and images for: Popular media records reveal multi-decadal trends in recreational fishing catch rates
Source: PLoS One. 2017 Aug 4;12(8):e0182345. doi: 10.1371/journal.pone.0182345 (PMC5544183; doi:10.1371/journal.pone.0182345)

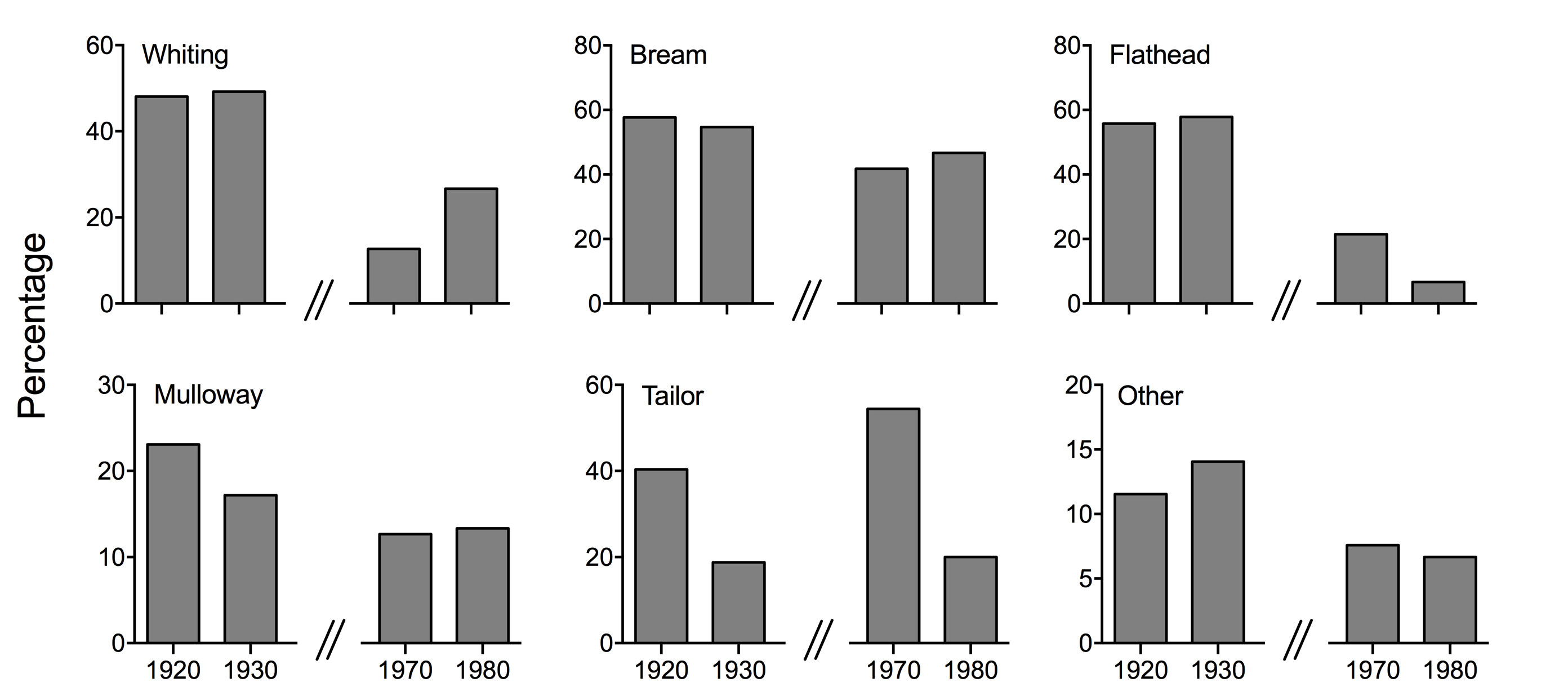

Supplement: S1 Fig — Percentage of catch records reporting to species that mentioned the occurrence of either whiting, bream, flathead, mulloway, tailor or other species. Decades where <10 records mentioned specific species in their catch are not included. (TIFF) [file pone.0182345.s001.tiff]
