# Supplementary material for: Popular media records reveal multi-decadal trends in recreational fishing catch rates
Source: PLoS One. 2017 Aug 4;12(8):e0182345. doi: 10.1371/journal.pone.0182345 (PMC5544183; doi:10.1371/journal.pone.0182345)
Supplement: S1 Table — (PDF) [file pone.0182345.s002.pdf]

Archival source	Location sourced	Years searched
Department of Harbours and Marine Annual Reports	State Library of Queensland	1892–1970
Fish Board Annual Reports	State Library of Queensland	1937–1981
Department of Agriculture and Fisheries landings records	Queensland Department of Agriculture and Fisheries	1990–2014
Fishery Inspectors' correspondence	Queensland State Archives	1910–1965
The Brisbane Courier	National Library of Australia	1884–1933
Courier Mail	National Library of Australia	1933–1954
The Telegraph	National Library of Australia	1872–1947
Sunday Mail	National Library of Australia	1926–1954
Gympie Times and Mary River Mining Gazette	National Library of Australia	1863–1919
The Maryborough Chronicle	National Library of Australia	1860–1954
Nambour Chronicle and North Coast Advertiser	Nambour Library	1903–1983
Noosa Advocate	Pomona Museum	1917–1933
Noosa News	Noosa Library and State Library of Queensland	1968–2015 (sub-sampled 1968–1990, 1 year every 3 years)
