# Supplementary material for: Popular media records reveal multi-decadal trends in recreational fishing catch rates
Source: PLoS One. 2017 Aug 4;12(8):e0182345. doi: 10.1371/journal.pone.0182345 (PMC5544183; doi:10.1371/journal.pone.0182345)
Supplement: S2 Table — (PDF) [file pone.0182345.s003.pdf]

**Response**	**Model estimate**	**SE**	**t-value**	**χ^2**	**df**	**p-value**
Mean competition catch rate	−0.003	0.001	−4.710	10.748	1	**0.001**
Best competition catch rate	−0.001	0.001	−1. 498	1.699	1	0.192
Mean competition weight per person	-0.0001	0.0001	-1.245	1.468	1	0.226
Best competition weight	-0.0002	0.0006	-0.359	1.125	1	0.724
